# A scoping review of generative AI and digital twins in tackling loneliness during hospital-to-home transitions

**DOI:** 10.3389/fdgth.2026.1802931

**Published:** 2026-09-28

**Authors:** Yuqing Zhang, Shengfeng Qin

**Affiliations:** Smart Design Lab, School of Design, Arts and Creative Industries, Northumbria University, Newcastle upon Tyne, United Kingdom

**Keywords:** digital twins, generative AI, hospital-to-home transitions, older adults loneliness, smart service design

## Abstract

Loneliness is an important psychosocial concern for older adults and may be intensified during the transition from hospital to home, when established routines, professional contact, functional capacity, and social participation can change simultaneously. This scoping review examined how generative artificial intelligence (GenAI), AI-enabled technologies, and digital twins (DTs) have been investigated in relation to loneliness, social connectedness, and psychosocial support for older adults, with particular attention to their relevance to hospital-to-home transitions. Following Joanna Briggs Institute guidance and PRISMA-ScR reporting principles, six interdisciplinary databases were searched for English-language peer-reviewed studies published between 2020 and 2026. Thirty-two primary studies were included in the final synthesis. Review-level publications were excluded from the primary synthesis and used separately to contextualize the broader technology landscape. The evidence revealed two largely separate research streams. Loneliness-focused studies predominantly investigated conversational agents, smart speakers, voice assistants, social and companion robots, large-language-model-supported systems, generative reminiscence, and AI-based loneliness detection, mainly in community, home, and long-term-care settings. In contrast, hospital-to-home studies primarily addressed continuity of care, rehabilitation, symptom communication, monitoring, personalized assistance, and service coordination, with loneliness rarely assessed as a primary outcome. Digital-twin applications relevant to loneliness were particularly limited and remained largely conceptual or design-oriented. No included study evaluated an integrated GenAI–DT intervention that continuously interpreted changing post-discharge context and used this information to personalize relational support within an accountable hospital-to-home care pathway. The principal gap is therefore not the absence of AI-enabled support, but the lack of integration between relational GenAI capabilities, context-aware sensing and digital-twin representations, and transitional-care services. Based on these findings, this review proposes a Relational Intelligence Ecosystem as an evidence-derived and theory-informed framework to guide the future design and evaluation of personalized, context-aware, and human-supervised smart services for older adults during hospital-to-home recovery.

## 1 Introduction

Global population ageing is accelerating. The number of people aged 60 years and older was estimated at approximately 727 million in 2020 (1) and is projected to reach 2.2 billion by 2070 (2). Supporting later life therefore requires attention not only to physical health and independence but also to social relationships and emotional wellbeing. The hospital-to-home transition is a particularly vulnerable period for older adults, as changes in care continuity, daily routines, and social contact can create or intensify loneliness-related challenges (3). Addressing these challenges therefore requires services that are both effective and responsive to individual needs and circumstances.

Loneliness is commonly defined as distress arising from a perceived discrepancy between desired and actual social relationships in terms of either quantity or quality (4). In later life, loneliness is associated with poorer physical and mental health, reduced quality of life, and increased risks of adverse outcomes, including mortality and cognitive decline (4–7). Because the drivers of loneliness vary across individuals, simply increasing the frequency of contact may not be sufficient. Interventions need to respond to each person's circumstances, preferences, capabilities, and unmet relational needs, making personalization particularly important.

Although returning home is often welcomed, the transition following hospitalization can be a difficult period of readjustment for older adults. Resuming daily life may be accompanied by uncertainty, worry, loneliness, and fear of managing symptoms or adverse events at home (8–10). At the same time, the routines, reassurance, and regular professional contact available in hospital are abruptly reduced. Qualitative studies describe the hospital-to-home transition as involving both relief and insecurity, including anxiety, isolation, disrupted continuity, and concerns about coping independently (3, 8, 9). Loneliness may therefore emerge or intensify through the interaction of physical vulnerability, reduced social participation, disrupted routines, and loss of dependable relational support. Earlier review papers show that hospitalization can create functional vulnerability and that successful hospital-to-home support depends on structured transition planning, continuity, and follow-up (11–14).

Digital health technologies, including telehealth, remote monitoring, and mobile applications, are increasingly used to support older adults during hospital-to-home transitions (15). However, these services do not always account for differences in digital capability, preferences, access, or home circumstances, which can limit engagement and the extent to which support reaches intended users. Wider digital-health research shows a similar pattern. Customized technologies have been developed to support communication, participation, belonging, companionship, and feelings of being seen, yet many studies emphasize usability and acceptance rather than directly evaluating loneliness outcomes (16–20). Technology alone therefore does not create meaningful connection; personalization, interaction quality, human support, and service integration remain important. These technology-enabled interventions include supported discharge and remote monitoring, virtual coaching, voice-assisted home care, smart-home support, and intelligent rehabilitations technologies spanning across clinical, functional, and engagement functions rather than loneliness alone (21–25).

Although Artificial intelligence is a key promising technology-enabling conventional digital interventions for loneliness, but existing reviews report inconsistent findings, with substantial variation in populations, intervention designs, settings, and outcome measures (26–35). More recently, generative AI, particularly large language models, has enabled more flexible natural-language interaction and the great capability of generating contents based on prompt inputs, having a potential to support personalized content, including conversational check-ins, reminders, explanations, reminiscence activities, and suggestions for social engagement. Empirical and design studies have begun to apply these capabilities to loneliness-related support for older adults, but the evidence remains heterogeneous, and concerns persist regarding privacy, trust, user control, and safe use (33–35). There, a further exploration of how generative AI can tailor its responses effectively based on individual user's needs and changing contexts which involve implementation, patient and public involvement, accountability, and alignment with users’ needs (36–40).

Digital twins may provide a complementary capability. A digital twin is a dynamic virtual representation of a person, environment, or system that is updated using real-world data. In healthcare, a human digital twin may integrate clinical, behavioral, social, and environmental information from health records, self-reports, wearables, smart-home sensors, and interaction histories (41–43). Such a representation could help identify changes in mobility, routines, communication, social participation, or self-reported wellbeing over time. Nevertheless, digital twins in older-adult home care remain an emerging approach. Current applications concentrate largely on monitoring and descriptive analytics, while predictive, personalized, and context-aware applications remain comparatively limited.

Generative AI and human digital twins may therefore address complementary aspects of personalized support. A digital twin could provide an evolving representation of the older adult's health, behavior, preferences, and home context, while generative AI could use relevant contextual information to produce timely and accessible responses. Depending on the service design, these responses could include a conversational check-in, clarification of discharge information, a personalized activity suggestion, facilitation of family contact, or escalation to a caregiver or professional. The potential value of this combination lies not in replacing human care, but in linking context-sensitive information to appropriate relational and service responses.

Existing review-level evidence illustrates this fragmentation (Supplementary Table 1). Reviews addressing loneliness and social connectedness among older adults have predominantly examined conversational AI, voice assistants, chatbots, social and companion robots, and other AI-enabled interaction technologies. Among the 11 reviews summarized in Supplementary Table 1, only one specifically addressed Hospital-at-Home: Yahya et al. examined digital-twin-enabling technologies for frailty management, focusing primarily on sensing, monitoring, risk prediction, personalized care, and clinical decision support. In contrast, the reviews focused on loneliness and social connectedness did not specifically examine Hospital-at-Home pathways. Collectively, these reviews demonstrate that AI-enabled relational support and digital-twin-enabled home care have largely developed as separate evidence streams, with no existing review integrating loneliness, generative AI, digital twins, and Hospital-at-Home or hospital-to-home transitional care within a single synthesis (16, 26–34, 41).

The evidence remains fragmented across these areas. Transitional-care research demonstrates the importance of continuity, reassurance, and coordinated support after discharge (44); generative-AI research increasingly explores conversational and relational interventions for older adults; and digital-twin research primarily addresses monitoring, representation, and personalization. Early concept-level work has begun to combine generative AI and digital-twin capabilities, but the reviewed literature did not identify an empirically validated, pathway-integrated smart service that combines these technologies to address loneliness among older adults during the hospital-to-home transition. The key gap is therefore not the absence of generative-AI interventions, but the lack of evidence showing how generative AI and digital twins can be integrated within an accountable transitional-care service to identify changing needs and deliver appropriate personalized support. Broader recovery evidence also addresses quality-of-life recovery, telerehabilitation, and the multidimensional mobility factors that shape successful return home (45–47).

A scoping review was selected because this is an emerging and interdisciplinary field encompassing heterogeneous evidence from gerontology, nursing, healthcare, human-computer interaction, service design, artificial intelligence, and engineering. The literature includes qualitative studies, prototypes, feasibility evaluations, trials, reviews, and conceptual architectures. A scoping approach is therefore appropriate for mapping the range and characteristics of existing evidence, clarifying current technological and service capabilities, and identifying the gaps that need to be addressed before an integrated smart service can be developed and evaluated in hospital-to-home care. Evidence from other care transitions illustrates that caregiver roles and emotions can also be destabilized when care settings change, although this is not direct hospital-to-home evidence (48). Qualitative work with lower-limb amputees and family caregivers further illustrates how functional dependency and caregiver needs can shape recovery and participation at home (49). These concerns sit within wider calls for person-centered geriatric care and healthy-ageing policy (50, 51).

Accordingly, the review was guided by three research questions:

1. What generative AI, AI-enabled, and digital twin technologies have been investigated for addressing loneliness, social isolation, or related psychosocial needs among older adults, and in what care contexts have they been studied?
2. What does current state-of-the-art research report about technological or service-based approaches addressing these needs and alleviating post-hospital loneliness?
3. What research gaps exist in the design and development of personalized, context-aware, and smart interventions to mitigate loneliness and enhance wellbeing during the hospital-to-home transition?

By synthesizing evidence across transitional care, AI-enabled loneliness interventions, and digital-twin research, this review aims to clarify the current state of the field and the specific gap at their intersection. Based on the evidence and gaps identified, it also proposes a conceptual framework and a development roadmap to guide future research and the design, integration, and evaluation of personalized smart services for older adults during hospital-to-home transitions. A service perspective is also consistent with scholarship arguing that smart technologies create value through coordinated service journeys rather than isolated devices (52).

## 2 Method

This scoping review explores the existing body of literature on smart and digital service technologies that aim to alleviate loneliness, enhance emotional wellbeing, and support hospital-to-home transitions among older adults. The review was designed to capture and map how generative AI and digital twins have been studied in relation to post-hospitalization recovery, social connection, and mental wellbeing between 2020 and 2026.

This study followed the Joanna Briggs Institute (JBI) methodology for scoping reviews and is reported in accordance with the PRISMA-ScR (Preferred Reporting Items for Systematic Reviews and Meta-Analyses extension for Scoping Reviews) checklist (53, 54). The review approach is conceptually grounded in the scoping review framework proposed by Arksey and O’Malley and further refined by Levac et al. to strengthen analytical depth and methodological clarity (55, 56). This methodological combination enables systematic mapping of heterogeneous evidence across multiple disciplinary domains, including healthcare, engineering, and human–computer interaction.

### 2.1 Identifying relevant studies

In line with JBI guidance, the Population–Concept–Context (PCC) framework was used to define the scope and eligibility criteria of this review (53). The population was defined as older adults aged ≥60 years. The concept focused on smart and digital service technologies designed to alleviate loneliness, enhance emotional wellbeing, or support psychosocial adaptation, and the context included hospital discharge, transitional care, and home-based/community recovery environments.

This framework guided both the formulation of research questions and the evidence selection. Rather than evaluating intervention effectiveness quantitatively, the review aimed to identify thematic patterns, design opportunities, and research gaps across multiple technological domains, reflecting the broad and rapidly evolving nature of this field.

The publication period was restricted to 2020–2026 to capture the contemporary phase of rapidly evolving intelligent and data-driven health technologies relevant to the aims of this review. The year 2020 represents an appropriate methodological boundary because it coincides with major advances in large-scale transformer-based language models and the emergence of increasingly adaptive, generative, and conversational AI capabilities, which subsequently accelerated with the public availability of ChatGPT and related generative AI systems. During the same period, digital twin research in healthcare also expanded substantially from predominantly conceptual applications toward more patient-centered, data-integrated, and service-oriented use cases. Restricting the review to this recent period therefore reduced conceptual and technological heterogeneity associated with earlier forms of artificial intelligence, rule-based conversational systems, and less mature digital-health infrastructures. This timeframe was considered appropriate for maintaining the currency and comparability of evidence in a rapidly evolving field while capturing developments most relevant to contemporary smart-service design.

Given that research related to health technologies for older adults spans diverse disciplines (e.g., design, psychology, human–computer interaction, nursing, and engineering), the search strategy targeted six databases to ensure interdisciplinary coverage: Scopus, Web of Science, PubMed, CINAHL, IEEE Xplore, and the ACM Digital Library. The search was restricted to the title and abstract fields, where supported by the database interface. The core search concepts were:

loneliness
AND (“older adult*” OR “older person*” OR “older people” OR “older individual*” OR elder* OR ageing OR aging OR senior*)
AND (“generative artificial intelligence” OR “generative AI” OR GenAI OR AI OR chatbot* OR “large language model*” OR LLM* OR ChatGPT OR “digital twin*”)

### 2.2 Study selection

All retrieved records were exported into Rayyan and Zotero reference management software, where both automatic and manual procedures were applied to remove duplicates. Screening occurred in two stages (1) title/abstract and (2) full-text following PRISMA-ScR guidance (54). Title and abstract screening were initially performed to exclude clearly irrelevant records, such as those focused on non-older adult populations, unrelated technology domains, or non-health contexts. Full-text screening was then conducted to confirm final eligibility according to the predefined inclusion criteria.

A comprehensive search was conducted across six databases, PubMed (*n* = 125), CINAHL (*n* = 116), Web of Science (*n* = 263), Scopus (*n* = 197), IEEE Xplore (*n* = 54), and ACM Digital Library (*n* = 73) yielding a total 828 records. In addition, 4 records were identified through manual searches. including reference list checking, citation tracking, and relevant supplementary sources, consistent with established scoping review procedures (55, 56). After automated and manual de-duplication, 562 unique records were retained for screening. Title screening excluded 441 records that clearly did not meet the inclusion criteria, leaving 121 records for abstract assessment. Following abstract screening, 104 articles progressed to full-text review to confirm eligibility. Following full-text screening and final eligibility reconciliation, 32 primary studies were included for detailed data extraction and thematic analysis. Review papers used for contextualization were handled separately and summarized in Supplementary Table 1.

The complete identification and selection process, from 832 total records (828 from databases + 4 from other sources) to 32 included studies, is illustrated in the PRISMA-ScR flow diagram (Figure 1) (54).

**Figure 1:**
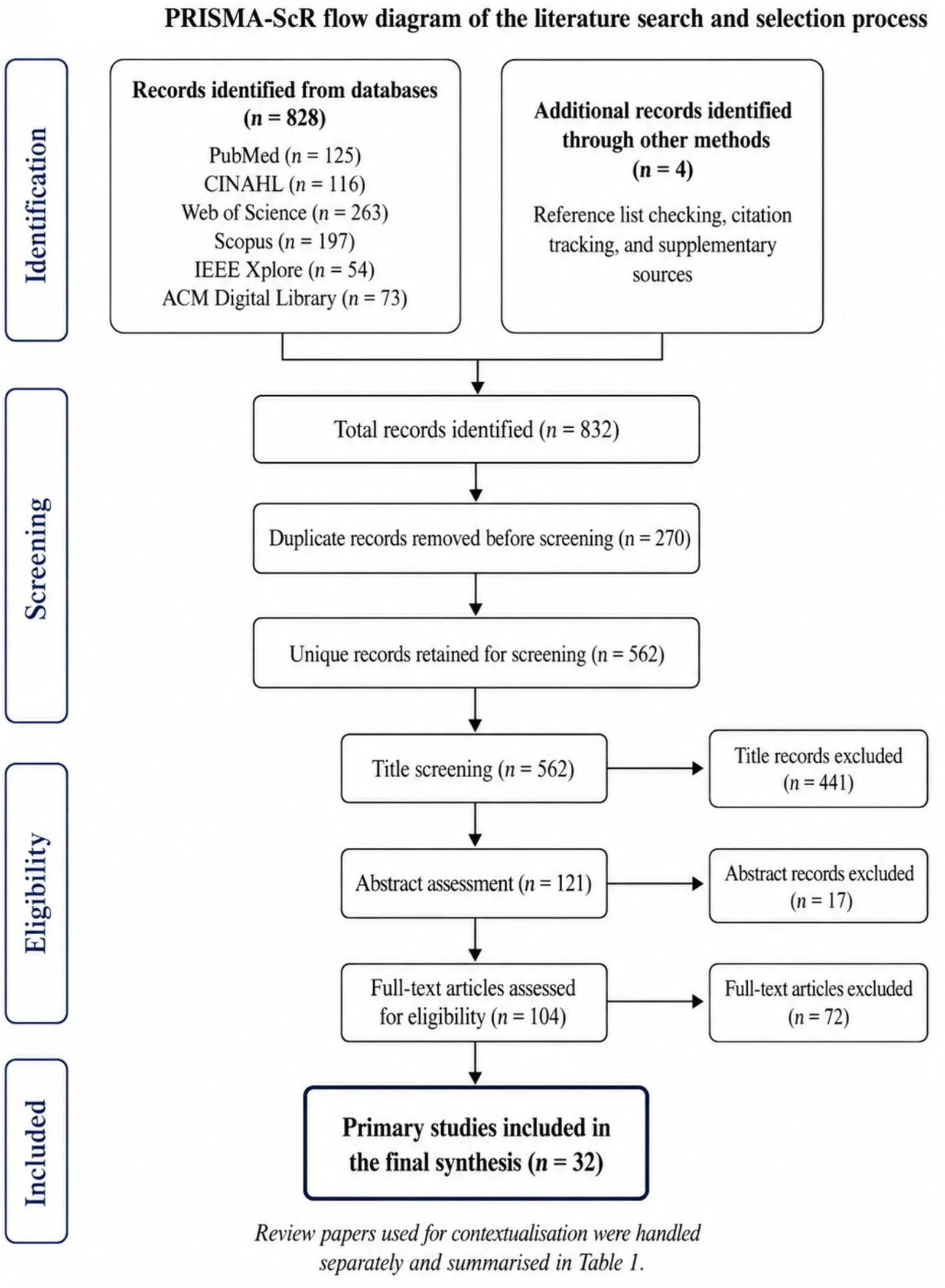
Flow diagram of the literature search and selection process (adapted from PRISMA).

Inclusion criteria:

- Older adults aged ≥ 60 years.
- Studies examining or designing smart or digital service technologies—including AI, digital twins, IoT, telehealth, or related solutions—aimed at alleviating loneliness or improving emotional wellbeing.
- Peer-reviewed studies published in English between 2020 and 2026.

Exclusion criteria:

Studies were excluded if they:

- Focused on populations younger than 60 years.
- Lacked an intervention or conceptual framework involving digital or smart technologies.
- Were grey literature, opinion papers, or non-peer-reviewed reports.
- Review articles were excluded from the primary synthesis; relevant review-level publications were retained separately for contextualization and are summarized in Supplementary Table 1.

### 2.3 Data charting and extraction

Following study selection, the included literature was reviewed and information relevant to the research questions was extracted and organized for descriptive mapping and narrative synthesis, informed by Joanna Briggs Institute (JBI) guidance for scoping reviews (53). Given the heterogeneity of the evidence base, which included empirical studies, design and development studies, the extracted information focused on study characteristics and findings most relevant to the aims of this review.

For the review papers, information was summarized in Supplementary Table 1, including the source, years covered by the review, problem investigated, whether the hospital-to-home period was addressed, the purpose of the AI or digital twin intervention, and the types of AI or digital twin technologies covered. For the 32 included primary studies, relevant information was identified from the articles, including the technology investigated, study context, target population, and findings related to loneliness, social isolation, psychosocial wellbeing, and hospital-to-home support, where applicable.

No formal quality appraisal or risk-of-bias assessment was undertaken, as the purpose of this scoping review was to map the breadth, characteristics, and gaps in the available evidence rather than to estimate pooled intervention effectiveness.

### 2.4 Summarizing and answering the research questions

Once data charting was completed, we applied the research questions to the extracted evidence to guide synthesis. To address RQ1, we first mapped the technologies represented in the evidence and the care contexts in which they were studied. Specifically, we compared intervention types (e.g., social robots, voice assistants, digital companions, telehealth-enabled transitional care, and DT/GAI-enabled concepts), along with study and participant characteristics, outcomes assessed, and reported associations with loneliness, social connectedness, and emotional wellbeing. We then developed a narrative summary of study objectives and key findings, enabling cross-study comparison and identification of patterns in how digital technologies support psychosocial wellbeing during hospital-to-home transitions. This synthesis informed our interpretation of the emerging relationships between digital twins, generative AI, and older adults’ wellbeing, as well as the evidence gaps and design opportunities for future smart service development.

## 3 Result

### 3.1 Characteristics and composition of the evidence base

The final included set comprised 32 primary studies published between 2020 and 2026 (57–88). The evidence base was heterogeneous and included empirical intervention studies, qualitative and mixed-methods studies, design and development research and feasibility evaluations evidence. Review papers were used to contextualize technology categories and broader patterns in the field rather than as direct evidence of intervention effectiveness. Across the literature, the strongest empirical evidence concerned conversational agents, voice-based systems, and socially assistive or companion robots, while generative-AI and digital-twin applications were generally more recent and less mature.

The reviewed literature represented chatbots and conversational agents, smart speakers and voice assistants, social and companion robots, LLM-enabled companion systems, generative-AI reminiscence, natural-language-processing and explainable-AI approaches for loneliness detection, passive sensing, and machine-learning risk assessment (57–88). Digital-twin applications were particularly limited, with two studies explicitly applying a digital-twin concept to older-adult home or loneliness-related support (62, 75).

The evidence formed two complementary but only weakly connected strands. Transition-focused studies examined continuity of care, symptom communication, rehabilitation, frailty, recovery, and service coordination after hospitalization (57–59, 63–65, 72, 73, 78). AI-focused studies addressing loneliness or social connectedness were conducted mainly in home and community settings, among older adults living alone, and in assisted- or long-term-care environments (61, 68, 70, 74, 76, 77, 79–83, 85–88). Palace et al. evaluated an AI-driven voice companion in post-acute and long-term-care settings (79), but the study did not follow participants through a hospital-discharge-to-home pathway. No included study evaluated an integrated generative-AI and digital-twin intervention for loneliness across an actual hospital-to-home transition.

### 3.2 RQ1: technologies investigated and care contexts

#### 3.2.1 Technology landscape

Research on loneliness and older adults was concentrated in relational and conversational AI rather than digital twins. The literature represented chatbots and conversational agents, smart speakers and voice assistants, social and companion robots, LLM-enabled companion systems, generative-AI reminiscence, natural-language-processing and explainable-AI approaches for loneliness detection, passive sensing, and machine-learning risk assessment (57–88). Generative-AI applications included ChatGPT-based interaction, LLM-supported companion robots, and personalized reminiscence, whereas digital-twin applications remained limited (62, 68, 75, 77, 85, 88).

The empirical evidence was strong for established conversational and robotic technologies. Smart-speaker and voice-assistant studies examined older adults living alone or using the systems at home (61, 70, 74), while social- and companion-robot studies included older adults living alone, community participants, and outpatient populations (81, 82, 85, 86, 88). More recent generative-AI studies included exploratory ChatGPT use (68), LLM-supported companion robots (85, 88), and AI-generated reminiscence content in long-term care (77). Other studies used language, behavioral, or sensor data to identify loneliness-related patterns or estimate loneliness risk rather than to provide companionship directly (60, 69, 71, 84).

#### 3.2.2 Care contexts represented in the evidence

Most empirical AI-focused studies were conducted outside hospital-to-home transitions. They were mainly situated in home and community contexts, among older adults living alone, or in assisted- and long-term-care environments (61, 68, 70, 74, 77, 79–83, 85–88). Palace et al. evaluated an AI-driven voice companion in post-acute and long-term-care settings (79), but this study did not follow older adults through a hospital-discharge-to-home pathway.

Transition-focused studies examined nurse-led follow-up after hospitalization (57), preferences for technology-mediated symptom communication after discharge (58), personalized assistive-care platforms (59), technologically monitored transitional care (63), co-design of a digital companion between hospital care and cardiac rehabilitation (64), AI-supported transitional-care needs and pathways (72, 73), and home-recovery programmers (65, 78). These studies establish the clinical, service, and recovery context of transition, but they do not provide direct evidence that generative AI or digital twins reduce loneliness during hospital-to-home recovery.

#### 3.2.3 RQ1 synthesis

Taken together, RQ1 identifies a clear context–technology mismatch. AI-based loneliness research is concentrated in community, home, and residential-care settings, whereas hospital-to-home studies primarily address continuity, recovery, communication, and care coordination. Digital twins are the least represented technology in the loneliness-focused evidence. No evaluated intervention in the reviewed literature combined generative AI and a digital twin to address loneliness across an actual hospital-to-home transition.

### 3.3 RQ2: AI- and digital-technology approaches relevant to loneliness

#### 3.3.1 Conversational AI, smart speakers, and voice assistants

The clearest empirical loneliness evidence came from conversational and voice-based systems used outside hospital-to-home pathways. In a two-wave longitudinal analysis of 291 older adults living alone, frequent use of an AI smart speaker was associated with improved loneliness at two-month follow-up, while depression decreased among both frequent and intermittent users (61). In a 12-week study of 50 older adults aged 60 years or older who lived alone with chronic musculoskeletal pain, both groups used conversational voice-assistant routines twice daily; loneliness decreased significantly in the enhanced-routine group, and depression scores decreased in both intervention groups (74). These studies suggest that repeated, low-burden voice interaction may support psychosocial outcomes for some community-dwelling older adults, but neither study examined the immediate post-discharge period.

Evidence from explicitly generative systems was more recent and generally less mature. In an exploratory study, 20 adults aged 60 years or older completed three ChatGPT conversations over two weeks with pre/post UCLA Loneliness Scale assessment and qualitative interviews; participants generally described the system as easy to use, engaging, and emotionally supportive, but the small sample and short exposure period limit conclusions about effectiveness (68). A separate cross-sectional study of 127 older adults found moderate-to-strong agreement between ChatGPT-4-adapted versions of several mental-health questionnaires and established measures, including the six-item UCLA loneliness measure, indicating potential for conversational assessment rather than demonstrating a loneliness intervention effect (76).

The qualitative evidence clarified how conversational systems were being used. Eighteen empty-nest older adults described using AI chatbots for self-expression, perceived emotional care, cognitive or recreational stimulation, role-playing, informal counselling, and reconnection with offline and online social networks; the authors explicitly focused on patterns of engagement rather than effectiveness (80). Among 18 low-income older adults living alone, perceived benefits of an AI conversational agent included information support, communication assistance, memory support, positive affect, security, and satisfaction, with technical assistance and free access contributing to the experience (70). These studies indicate that the surrounding service conditions are part of the intervention experience.

Not all conversational-agent findings showed reduced isolation. In an eight-week, three-wave experiment involving 110 older adults living alone, perceived social presence of a dementia-prevention chatbot predicted greater interpersonal resilience, but its effect on isolation was not significant (83). A post-acute and long-term-care feasibility study enrolled 28 participants, of whom 23 completed four-week follow-up; the AI voice companion was associated with reductions in depression and anxiety, particularly among those with higher baseline symptom severity, but loneliness was not established as an outcome effect and participants were not followed through a hospital-to-home pathway (79). A three-month prospective study of 120 community-dwelling older adults living alone reported improvements in both anxiety and UCLA loneliness scores with an AI voice chatbot, providing stronger emerging intervention evidence but again outside the transition context (87).

#### 3.3.2 Social and companion robots

Robot-based studies showed both positive and null findings. In a two-month explanatory sequential mixed-methods study, 60 older adults living alone were allocated to an AI care-robot group (*n* = 30) or control group (*n* = 30); significant group-by-time effects were reported for social isolation, loneliness, and depression, while qualitative interviews identified social presence, emotional companionship, restoration of routine, and self-efficacy as perceived mechanisms (82). A small pilot study with 15 single older adults reported improvements over time in loneliness, quality of life, subjective happiness, perceived sociability, and technology acceptance after repeated interactions with the LOVOT robot, although the absence of a control group and the small sample limit causal inference (86).

Other robot studies were more cautious. Multimodal data from the Hyodol socially assistive robot identified three engagement personas; some clusters showed numerical reductions in loneliness and depression, but these changes were not statistically significant (81). In a UK-Japan qualitative study of an LLM-supported companion robot, community-dwelling participants in London (*n* = 17) and outpatients with mild cognitive impairment in Osaka (*n* = 8) valued potential companionship but raised concerns about latency, turn-taking, conversational depth, privacy, data governance, access, and substitution for human contact (85). A subsequent refinement study using a GPT-4o-enabled robot reduced median response latency and improved perceived usability and conversational naturalness, while questionnaire measures of companionship changed little (88). These findings separate technical conversational fluency from the stronger claim that an LLM system produces a sustained relational benefit.

#### 3.3.3 Generative AI beyond conversation

The literature also included emerging uses of generative AI that were not conventional chatbots. The HARMONEE pilot used AI-generated images based on participants’ memories to support personalized reminiscence in two long-term-care facilities. Among 34 participants over 60 days, retention was 85%, all participants completing the satisfaction assessment reported high satisfaction, and short-term mood improved significantly; qualitative data described happiness, nostalgia, memory stimulation, and social connectedness (77). This study demonstrates a feasible generative-AI mechanism for personalized social and emotional engagement, but it did not evaluate hospital-to-home recovery or loneliness as the primary clinical endpoint.

#### 3.3.4 Loneliness detection, predictive AI, sensing, and digital twins

A separate group of studies used AI to identify loneliness-related patterns rather than to deliver companionship. In 80 older adults aged 66–94 years, natural-language-processing and machine-learning models predicted qualitative loneliness with 94% precision and quantitative loneliness with 76% precision from interview language (60). A later explainable-AI study of 97 older adults reported classification accuracy of 0.889 and an area under the curve of 0.80, with social-relationship and loneliness interview sections contributing strongly to prediction (69). A large cross-sectional study using data from approximately 16,000 Chinese adults aged 65 years or older developed several machine-learning models for loneliness-risk assessment and identified marital status as an important predictor (71). These studies address identification and risk stratification; they do not establish that prediction itself reduces loneliness.

The SMILE study extended this approach by combining passive sensing with personalized counterfactual pattern analysis. A six-month feasibility study included 18 participants in the United States and 15 in Japan. Its time-series model achieved 96.25% accuracy for loneliness detection, and a diffusion model generated counterfactual behavioral profiles intended to identify patterns associated with lower predicted loneliness (84). The study demonstrates technical feasibility for context-sensitive detection and explanation, but it did not test whether the resulting signals triggered an effective social or clinical intervention.

Digital-twin evidence was especially sparse. Hu et al. described a smart-home remote-collaboration system combining a digital twin, IoT data, mixed reality, and family participation to support remote assistance and companionship for empty-nest families (62). Zhang et al. proposed a smart flowerpot integrating IoT sensing, a user digital twin, and generative AI to personalize loneliness-related interactions; qualitative evaluation suggested engagement and wellbeing potential, but the work remained a concept/design evaluation rather than a controlled loneliness intervention (75). Neither study was conducted during hospital-to-home transition, and no longitudinal study in the reviewed evidence evaluated a continuously updated digital twin linked to a generative relational agent across discharge and home recovery.

### 3.4 RQ3: evidence gaps for personalized, context-aware smart services

#### 3.4.1 Gap 1: the transition and loneliness evidence bases remain largely separate

The most important gap was contextual. The transition-focused literature documents uncertainty, loss of support, changing routines, functional limitations, and the need for coordinated follow-up (3, 8, 57, 58, 63–65, 72, 73). AI-focused studies, in contrast, evaluate loneliness or social connectedness mainly among people living alone, community-dwelling older adults, retirement-home residents, or long-term-care populations (61, 68, 70, 74, 76, 77, 79–83, 85–88). No included study explicitly evaluated generative AI or a digital twin throughout a hospital-to-home pathway. Therefore, transfer of community-based findings to the immediate post-discharge period remains an untested assumption.

#### 3.4.2 Gap 2: loneliness is not aligned with transitional-care outcome measurement

The transition studies prioritized length of stay, readmission, frailty, functional recovery, rehabilitation participation, communication, care coordination, or quality of life (58, 63–65, 72, 73, 78). By contrast, several AI studies used validated loneliness measures, including versions of the UCLA Loneliness Scale (60, 68, 76, 87). This outcome mismatch prevents direct assessment of whether better transitional continuity actually changes loneliness, or whether reductions in loneliness influence recovery, service use, or readmission.

#### 3.4.3 Gap 3: generative AI and digital twins are at different levels of maturity

The evidence should not present generative AI and digital twins as equally established interventions. Conversational AI and socially assistive robots already include longitudinal, experimental, qualitative, and feasibility studies, although findings remain heterogeneous (61, 68, 70, 74, 76, 77, 79–83, 85–88). In contrast, the two digital-twin records were design-oriented and did not demonstrate loneliness reduction (62, 75). GenAI-specific work was also concentrated in recent exploratory studies, design papers, protocols, and short deployments. The evidence therefore supports feasibility and design potential more strongly than long-term effectiveness.

#### 3.4.4 Gap 4: detection, personalization, and relational response are rarely connected

Predictive and sensing studies can identify loneliness-related language, behavior, or risk profiles (60, 69, 84), while conversational and robotic systems can provide interaction and social presence (61, 68, 70, 74, 76, 77, 79–83, 85–88). However, the reviewed evidence rarely linked these functions in a closed service pathway. No included study demonstrated a sequence in which changing post-discharge context was detected, interpreted, used to personalize a relational response, and escalated to family or professionals when needed. This missing connection is central to the proposed combination of digital twins and generative AI.

#### 3.4.5 Gap 5: implementation conditions and governance affect whether relational technologies are usable

Across studies, adoption depended on more than technical performance. Older adults raised issues of usability, response latency, user control, conversational depth, AI literacy, privacy, data governance, affordability, and concern that AI might substitute for human contact (17–20, 70, 85, 88). The evidence also showed that technical assistance and low-cost or free access could improve the experience of use (70). These findings indicate that onboarding, accessibility, human oversight, and escalation arrangements should be treated as components of a smart service rather than as secondary deployment considerations.

### 3.5 Evidence-derived functional themes informing the subsequent framework

Comparison of the transition and AI studies produced four recurring functions. First, service continuity and escalation concerned follow-up contact, coordination, accountability, and access to human help after discharge (57, 58, 64, 72, 73). Second, home-context enablement concerned practical access, usability, technical assistance, routines, and the capacity to sustain use (17–20, 65, 70, 85, 88). Third, adaptive sensing and personalization concerned detection of changing needs through monitoring, language analysis, sensor data, predictive models, or digital representations (60, 62, 69, 75, 76, 84). Fourth, relational interaction concerned conversation, reassurance, companionship, social presence, reminiscence, and support for reconnecting with other people (61, 68, 70, 74, 76, 77, 79–83, 85–88).

These four themes are reported as analytical groupings of functions observed across the literature, not as four technologies and not as empirically validated causal layers. No study in the reviewed evidence integrated all four functions within a single hospital-to-home intervention. Transition studies were strongest in continuity and service coordination; AI-companion studies were strongest in relational interaction; predictive AI and digital-twin studies were strongest in sensing and representation. The absence of an intervention that connects these functions is itself the principal synthesis finding and provides the empirical basis for discussing an integrated framework in the following section.

## 4 Discussion

### 4.1 Principal findings

This scoping review reveals a three-level evidence pattern. First, artificial-intelligence and generative-AI (GenAI) technologies are already being used to provide loneliness-related and psychosocial support for older adults. The evidence includes smart speakers and conversational voice systems, AI chatbots, social and companion robots, exploratory ChatGPT interaction, LLM-supported companion robots, and GenAI-enabled reminiscence (61, 68, 74, 77, 80, 82, 85, 88). These studies show that AI can already deliver forms of conversation, emotional expression, social presence, reminders, reminiscence, and personalized interaction. However, the strength of evidence varies substantially: some studies reported improvements in loneliness or related outcomes, whereas others were qualitative, feasibility-oriented, focused on acceptability, or found non-significant effects (79, 81, 83, 85, 88). GenAI should therefore be regarded as an emerging intervention modality with demonstrated feasibility and promising relational functions, rather than as a consistently effective treatment for loneliness.

Second, the literature has begun to explore the combination of GenAI with digital-twin (DT) concepts, but this integration remains at an early design stage. Importantly, the evidence is not completely absent. Zhang et al. (75) explicitly combined GenAI, a user digital twin, IoT sensing, and a reconfigurable smart flowerpot to personalize loneliness-related interaction for older adults living alone. Hu et al. (62) separately used a digital twin with smart-home and mixed-reality technologies to support remote family collaboration and companionship. These studies demonstrate the conceptual and technical possibility of combining contextual representation with relational or social support. Nevertheless, they do not establish the effectiveness of a mature GenAI-DT smart service: evaluation was limited, the interventions were not embedded in routine care pathways, and neither study tested longitudinal loneliness outcomes during post-hospital recovery.

Third, the specific hospital-to-home scenario remains largely unaddressed. Transition-focused studies in this review examined continuity, symptom communication, rehabilitation, frailty, personalized digital assistance, and care coordination after discharge (57–59, 63–65, 72, 73, 78). In parallel, loneliness-focused AI studies were conducted mainly among community-dwelling older adults, people living alone, care-home residents, or long-term-care populations. The key gap is therefore not an absence of AI innovation, but a translational and integration gap: no included study demonstrated a pathway-integrated smart service in which GenAI and a digital-twin or comparable context model were jointly used to identify changing needs, personalize relational support, and connect that support to accountable human care during the hospital-to-home transition.

This distinction is central to the contribution of the review. The literature already provides evidence for individual capabilities - relational interaction through AI and GenAI, contextual sensing and prediction, early digital-twin representations, and digitally supported transitional care - but these capabilities remain fragmented. The research opportunity is to determine whether they can be responsibly orchestrated into a coherent smart service for older adults who are particularly vulnerable to loneliness, uncertainty, and loss of support after hospital discharge.

### 4.2 Interpretation of findings

#### 4.2.1 Generative AI already provides relational intervention capabilities

The newer GenAI studies extend earlier digital companionship research by moving beyond fixed scripts toward more flexible and personalized content. The exploratory ChatGPT study used repeated conversations with older adults and reported that participants experienced the system as engaging and emotionally supportive (68). LLM-supported companion-robot studies showed that older adults could perceive social presence and value conversational flexibility, while also identifying limitations in conversational depth, response timing, user control, privacy, and the risk of substituting AI for human contact (85, 88). The HARMONEE study used GenAI-generated images to support personalized reminiscence and reported high satisfaction, improved short-term mood, and qualitative experiences of nostalgia, memory stimulation, and social connectedness (77). Together, these studies indicate that GenAI can already function as an intervention-delivery mechanism for relational and psychosocial support, although robust evidence for sustained loneliness reduction remains limited.

The intervention value of GenAI appears to arise from the relational functions it can deliver rather than from generative capability itself. Across the broader AI literature, older adults valued opportunities for self-expression, emotional reassurance, perceived companionship, routine support, information assistance, and reconnection with other people (70, 80, 82). Conversely, greater conversational fluency did not necessarily produce stronger companionship (88), and perceived chatbot social presence did not necessarily reduce social isolation (83). These findings suggest that natural language generation is a means of delivering support; it is not, by itself, the mechanism that resolves loneliness. Relational benefits depend on the relevance, timing, continuity, and social meaning of the interaction.

#### 4.2.2 Digital twins can provide context, but context must lead to action

The potential contribution of digital twins is different. A DT is most relevant not as a companion, but as a dynamic representation of the older adult and the changing home-recovery context. The reviewed DT and sensing studies illustrate the kinds of information that may contribute to such representation, including environmental data, routines, behavioral patterns, language, and loneliness-related risk indicators (60, 62, 69, 71, 75, 84). In principle, these data could help distinguish different reasons for apparent social withdrawal - for example, reduced mobility, fatigue, disrupted routines, reduced communication, or a change in emotional state - and thereby support more context-sensitive intervention.

However, prediction or representation is not equivalent to care. The sensing and machine-learning studies mainly addressed detection or risk estimation rather than intervention (60, 69, 71, 84). Similarly, the DT studies demonstrated technical or design possibilities but did not establish a closed service loop in which a detected change reliably generated an appropriate response. The important design question is therefore what happens after the system identifies a need. A context model becomes clinically and socially meaningful only when its inference is translated into a proportionate action, such as a conversational check-in, a personalized activity or reminiscence prompt, facilitation of family contact, adjustment of follow-up, or escalation to a caregiver or professional.

#### 4.2.3 Hospital-to-home transition is a distinct service context

The hospital-to-home transition makes this integration particularly important because older adults may experience several forms of vulnerability at the same time. Transition studies describe uncertainty about symptoms and recovery, reduced physical capacity, disrupted routines, changing care responsibilities, and reduced access to the reassurance available in hospital (57, 58, 63–65, 72, 73, 78). Loneliness in this period therefore cannot be treated only as a lack of conversation. It may be intertwined with functional limitations, confidence, access to help, caregiver availability, and the ability to re-establish everyday social participation.

For this reason, a GenAI companion deployed as a standalone application would address only part of the problem. Likewise, a DT or sensor platform that identifies behavioral change without a relational or service response would provide information without support. A hospital-to-home smart service must connect the two: context awareness should inform the timing and form of relational intervention, while the relational interface should remain connected to human care, family networks, and escalation pathways. This requirement differentiates a transitional smart service from a general-purpose chatbot or home-monitoring system.

### 4.3 Proposed relational intelligence ecosystem framework

Building on these findings, this review proposes the Relational Intelligence Ecosystem as a forward-looking, theory-informed and evidence-derived framework for designing smart services that address loneliness during hospital-to-home transitions. The framework does not assume that GenAI and DTs are already validated as an integrated intervention. Instead, it organizes the separate capabilities identified in the literature into a service architecture that can be tested prospectively. Transitional-care theory provides the underlying emphasis on continuity, coordination, patient capacity, and support across settings (44), while the reviewed technology studies provide evidence for context sensing, personalization, relational interaction, and implementation requirements.

The framework comprises four interdependent functional layers. Their purpose is not to represent four separate technologies, but to ensure that the main functions identified across the evidence are connected within one accountable service. The four layers also provide a direct response to the fragmentation identified in this review.

#### 4.3.1 Layer 1 - care-transition service and continuity

The first layer provides the service backbone. It defines who remains responsible after discharge, what follow-up is expected, how the older adult can obtain help, and how concerns are escalated. Transition studies show that communication, follow-up, coordination, and accessible professional support are central to confidence and recovery after discharge (57–59, 63, 64, 72, 73). In the proposed ecosystem, GenAI and DT functions operate within this service structure rather than outside it. This layer prevents an intelligent system from becoming an isolated consumer tool with no clear accountability when needs exceed its role.

#### 4.3.2 Layer 2 - home-context enablement

The second layer concerns the conditions that make continued use possible in the home. It includes onboarding, accessible interaction, digital literacy support, affordability, caregiver participation, integration with routines, and appropriate sensing or self-report mechanisms. Studies of older adults’ technology use repeatedly show that usefulness, confidence, technical assistance, privacy expectations, and fit with everyday life influence sustained engagement (17–19, 65, 70, 85, 88). This layer is necessary because a highly personalized service cannot function if the intended user cannot access, understand, trust, or sustain it during recovery.

#### 4.3.3 Layer 3 - adaptive context intelligence and digital twin

The third layer represents the context-intelligence function. Here, a DT or comparable dynamic person-context model could integrate selected clinical, behavioral, social, environmental, and interaction data to represent how the older adult's situation changes over time. The reviewed DT, sensing, NLP, and risk-modelling studies show components of this capability (60, 62, 69, 71, 75, 84). Its purpose is not simply to accumulate data, but to identify meaningful changes and generate interpretable cues for personalization. For example, reduced interaction combined with lower activity and self-reported low mood may require a different response from reduced interaction caused by temporary fatigue after rehabilitation. The DT layer therefore provides the contextual bridge between the person's evolving circumstances and the intervention delivered.

#### 4.3.4 Layer 4 - relational GenAI intervention

The fourth layer translates contextual information into an interaction that the older adult can experience as useful and supportive. GenAI is positioned primarily in this layer because the reviewed evidence demonstrates its capacity for flexible dialogue, personalized prompts, reminiscence, emotional expression, information support, and conversational companionship (68, 77, 80, 85, 88). Depending on need and preference, the response could involve a brief check-in, explanation, motivational prompt, personalized activity suggestion, reminiscence interaction, facilitation of family contact, or a recommendation to seek human support. The aim is not to simulate a replacement relationship, but to provide low-burden relational support and to strengthen connection with the wider care and social network.

The four layers form a feedback loop rather than a linear technology stack. Service responsibilities established in Layer 1 shape what actions are permitted; Layer 2 determines whether the service can operate effectively in the person's home; Layer 3 updates the representation of needs and context; and Layer 4 delivers or facilitates a relational response. The outcome of that response - including engagement, non-response, expressed preference, or escalation - can then update the context model and inform subsequent service decisions. Human caregivers, family members, and professionals intersect with all four layers rather than sitting outside the system.

Governance, consent, privacy, transparency, equity, interoperability, and human oversight should be treated as cross-cutting requirements. They are not proposed as an additional layer because they constrain every part of the ecosystem. In particular, a DT-enabled service should apply data minimization and collect only information linked to a defined support purpose; GenAI outputs should be bounded by explicit safety rules; and users should know when they are interacting with AI, how personal information is used, and how to reach human support. The framework should therefore be interpreted as a testable service-design proposition, not as evidence that the four layers are already necessary and sufficient for reducing loneliness.

### 4.4 Roadmap for developing and evaluating the smart service

The evidence also supports a staged roadmap for moving from the current fragmented literature toward a pathway-integrated GenAI-DT smart service. The stages are sequential in development logic but should remain iterative, with findings from later testing feeding back into design and governance.

#### 4.4.1 Stage 1 - define transitional loneliness needs and co-design the service

The first stage should establish the target users, transition period, service responsibilities, and different pathways through which loneliness may arise after discharge. Older adults, caregivers, clinicians, transitional-care staff, and service designers should be involved in defining meaningful support scenarios, acceptable data use, escalation boundaries, and preferred modes of interaction. This stage should produce a service blueprint and a minimum data specification rather than beginning with a technology-led assumption that more sensing or more conversation is inherently beneficial.

#### 4.4.2 Stage 2 - validate GenAI and context-intelligence components separately

The second stage should test the two core technical capabilities before full integration. GenAI components should be evaluated for conversational relevance, accessibility, relational quality, inappropriate responses, user control, and ability to support rather than displace human connection. DT or context-intelligence components should be evaluated for the accuracy, interpretability, and usefulness of the information they represent or infer. The smart-flowerpot study (75) provides an early proof of concept for connecting GenAI, IoT, and a user digital twin, but future work should demonstrate that the resulting personalization is meaningful to users and linked to measurable outcomes.

#### 4.4.3 Stage 3 - integrate the system within a real hospital-to-home pathway

The third stage should move beyond laboratory or stable community settings and test the integrated service across discharge and early recovery at home. The pilot should begin before or around discharge so that preferences, baseline loneliness, support networks, and relevant recovery information can be established with consent. At home, the service should test whether contextual information can appropriately modify GenAI support and whether predefined escalation routes function in practice. Feasibility outcomes should include engagement, usability, technical reliability, response appropriateness, escalation events, burden on users and caregivers, and reasons for non-use.

#### 4.4.4 Stage 4 - evaluate effectiveness, safety, and implementation

Once feasibility is established, comparative or hybrid effectiveness-implementation studies should evaluate whether the integrated service produces added value beyond usual transitional care or human follow-up alone. Loneliness and social connectedness should be measured directly using validated instruments alongside quality of life, confidence, functional recovery, service use, and relevant clinical outcomes. Safety evaluation should include misleading GenAI responses, false or missed risk signals, privacy or governance incidents, inappropriate escalation, dependency concerns, and effects on professional and caregiver workload. Process evaluation is essential for determining which component produced benefit, for whom, and under what circumstances.

#### 4.4.5 Stage 5 - scale, personalize, and govern for long-term use

The final stage should examine transferability across services, populations, cultures, and levels of digital access. Research should determine whether different transition profiles require different combinations or intensities of the four framework layers rather than a single fixed intervention. Long-term implementation will also require interoperability with existing care systems, sustainable service ownership, equitable alternatives for people unable or unwilling to use digital tools, and governance arrangements that can adapt as GenAI and DT technologies evolve.

### 4.5 Implications for smart-service design

The framework and roadmap shift the design focus from individual technologies to service orchestration. GenAI contributes most directly to relational intervention; DTs and associated sensing contribute to context representation and adaptive personalization; transitional-care services contribute continuity and accountability; and home-context enablement determines whether the system can be used in practice. None of these functions is sufficient alone. Their value depends on how they are connected around the older adult's changing needs and existing human relationships.

This service perspective also provides a more precise interpretation of personalization. Personalization should not mean simply changing the wording of an AI response. It should involve selecting whether an intervention is needed, what kind of support is appropriate, when it should be delivered, through which channel, and when the system should instead facilitate human contact. A DT-informed context model may help provide this situational information, while GenAI may help translate it into accessible interaction. The effectiveness of that combination, however, must be established empirically rather than assumed from technical capability.

### 4.6 Discussion summary

The reviewed evidence therefore supports a more specific conclusion than either technological optimism or technological absence. GenAI is already being explored as a means of delivering loneliness-related and psychosocial interventions to older adults, and early work demonstrates flexible conversation, companionship-oriented interaction, and personalized reminiscence. GenAI-DT integration has also begun at the concept level, most explicitly in the smart-flowerpot study (75), but it has not matured into a validated, pathway-integrated service. Most importantly, no included study demonstrated an integrated GenAI-DT smart service designed specifically for older adults transitioning from hospital to home. This is the central research gap identified by the review.

The proposed Relational Intelligence Ecosystem translates that gap into a testable framework by connecting care-transition continuity, home-context enablement, adaptive context intelligence, and relational GenAI intervention, with governance and human oversight operating across the system. The accompanying roadmap describes how this proposition can progress from co-design and component validation to pathway integration, effectiveness evaluation, and eventual scale-up. Together, the framework and roadmap provide a structured basis for future research without overstating the maturity of the current evidence.

## 5 Limitations

This scoping review has several limitations. First, the evidence directly located at the intersection of loneliness, advanced AI, digital twins, and hospital-to-home transition was sparse. To map this emerging field, the synthesis therefore brought together transition-specific studies with AI- and digital-twin studies conducted mainly in community, living-alone, residential-care, post-acute, and long-term-care settings. This approach is appropriate for identifying transferable functions and design opportunities, but it also means that mechanisms observed outside the discharge pathway cannot be assumed to operate in the same way during the immediate post-hospital recovery period.

Second, evidence maturity was uneven across technologies. Conversational agents, smart speakers, and social or care robots have begun to generate longitudinal, comparative, and mixed-method evidence, although findings remain heterogeneous (61, 74, 79, 81, 82, 87). In contrast, explicitly generative-AI interventions are still represented largely by exploratory, qualitative, feasibility, and early deployment studies, while digital-twin evidence relevant to loneliness is particularly limited (62, 68, 75, 77, 85). The identified GenAI-DT integration remains concept-level rather than a validated hospital-to-home service. Consequently, this review cannot establish the effectiveness, safety, comparative advantage, or long-term sustainability of an integrated GenAI-DT intervention.

Third, comparability was constrained by variation in study populations, intervention components, exposure periods, comparison conditions, and psychosocial outcomes. Loneliness was measured with validated instruments in some studies, whereas others relied on social isolation, depression, social presence, engagement, satisfaction, or qualitative accounts as related indicators. Short follow-up periods in several studies further limit conclusions about sustained use, habituation, dependency, or whether early improvements persist once novelty and research contact diminish. These differences precluded meaningful quantitative pooling and require caution when interpreting apparent intervention effects across technology categories.

Finally, the review was restricted to English-language, peer-reviewed literature within the specified publication period and databases. Given the pace of development in GenAI and digital-twin research, relevant prototypes, conference outputs, unpublished evaluations, or newly emerging systems may not have been captured. Terminology is also evolving rapidly, and similar capabilities may be described without using labels such as generative AI, large language model, or digital twin. In addition, much of the available evidence was generated in relatively well-resourced settings; therefore, transferability to different health systems, cultural contexts, levels of digital access, and older adults with greater cognitive, sensory, socioeconomic, or language-related barriers remains uncertain. In addition, non-peer-reviewed design work such as uSafe was retained in background reference checking but was not treated as included evidence under the stated peer-reviewed eligibility criterion (89).

## 6 Conclusion

This scoping review shows that the field has progressed beyond the absence of technological intervention. AI-enabled conversational agents, smart speakers, social and care robots, and emerging generative-AI systems are already being investigated as ways to provide companionship, emotional expression, reassurance, routine support, reminiscence, and other loneliness-related interventions for older adults (61, 68, 70, 74, 77, 79–83, 85–88). Digital twins offer a different but potentially complementary capability: representing changing clinical, behavioral, social, and home-context information that may support monitoring and personalization (62, 75). Early work has begun to combine generative AI with digital-twin concepts, but this integration remains at an exploratory or design stage rather than constituting a mature, validated smart service.

The central gap identified by this review is therefore a translational and integration gap. The literature provides evidence for GenAI-enabled relational intervention, separate evidence for context sensing and digital-twin approaches, and an established transitional-care need for continuity, reassurance, and accountable follow-up. However, no included study evaluated a pathway-integrated service in which a digital twin or comparable context-intelligence function continuously informs a generative-AI intervention for loneliness while remaining connected to human caregivers, professionals, and escalation pathways during the transition from hospital to home. The specific post-discharge context is important because recovery-related fatigue, reduced mobility, disrupted routines, changing care needs, and sudden loss of professional contact may alter both loneliness risk and the way an older adult can engage with technology.

To address this gap, the proposed Relational Intelligence Ecosystem brings together four complementary functions: care-transition continuity and escalation, home-context enablement, adaptive context intelligence and digital-twin representation, and relational GenAI intervention. Governance, privacy, equity, interoperability, consent, and human oversight operate across these functions. The accompanying roadmap translates the framework into a staged research programmer progressing from needs identification and co-design, through component validation and pathway integration, to effectiveness-implementation evaluation and longer-term scale-up. Neither the framework nor the roadmap should be interpreted as evidence that an integrated GenAI-DT service is already effective; rather, they specify a testable direction derived from the gaps and mechanisms mapped in this review.

Future research should therefore move from isolated technological demonstrations toward longitudinal, pathway-embedded evaluations that recruit older adults around hospital discharge, directly measure loneliness and social connectedness, compare technology-supported care with appropriate human and usual-care conditions, and report safety, equity, escalation, and implementation outcomes alongside clinical recovery. If these requirements can be met, integrating context-aware digital-twin capabilities with carefully governed generative-AI interaction may provide a route toward more personalized and relationally responsive smart services, while preserving rather than replacing the human relationships on which successful hospital-to-home recovery depends.
